# Linear and nonlinear optical properties of boron phosphide nanotubes: insights into third-harmonic generation and magneto-optical tunability

**DOI:** 10.1039/d5ra06145h

**Published:** 2025-11-06

**Authors:** Raad Chegel

**Affiliations:** a Department of Physics, Faculty of Science, Razi University Kermanshah Iran Raad.Chegel@gmail.com

## Abstract

Boron phosphide nanotubes (BPNTs) are promising materials for optoelectronic applications, yet their nonlinear optical (NLO) properties, crucial for advanced photonic technologies, remain largely unexplored. This paper presents a comprehensive theoretical investigation of the linear and NLO responses of zigzag (*n*, 0) BPNTs within the range 20 ≤ *n* ≤ 28, using a fifth nearest-neighbor tight-binding model combined with the density-matrix formalism. The effects of nanotube radius and an external axial magnetic field are systematically analyzed. The results reveal that increasing the nanotube radius induces systematic red-shifts of infrared absorption peaks and a unique blue-shift in the visible range. More significantly, an axial magnetic field, *via* the Aharonov–Bohm effect, lifts subband degeneracy, causing a predictable splitting of all optical peaks. This magneto-optical coupling leads to a dramatic and tunable enhancement of NLO phenomena, with the primary third-harmonic generation (THG) peak intensity increasing under magnetic fields. This study provides the first comprehensive account of NLO effects in BPNTs, establishing magnetic-field control as a powerful strategy for designing tunable nanophotonic devices like optical switches and frequency converters.

## Abstract 2

Zigzag boron-phosphide nanotubes show radius-dependent shifts in third-harmonic generation, while axial magnetic fields induce controllable splitting of the resonances.

## Graphical abstract

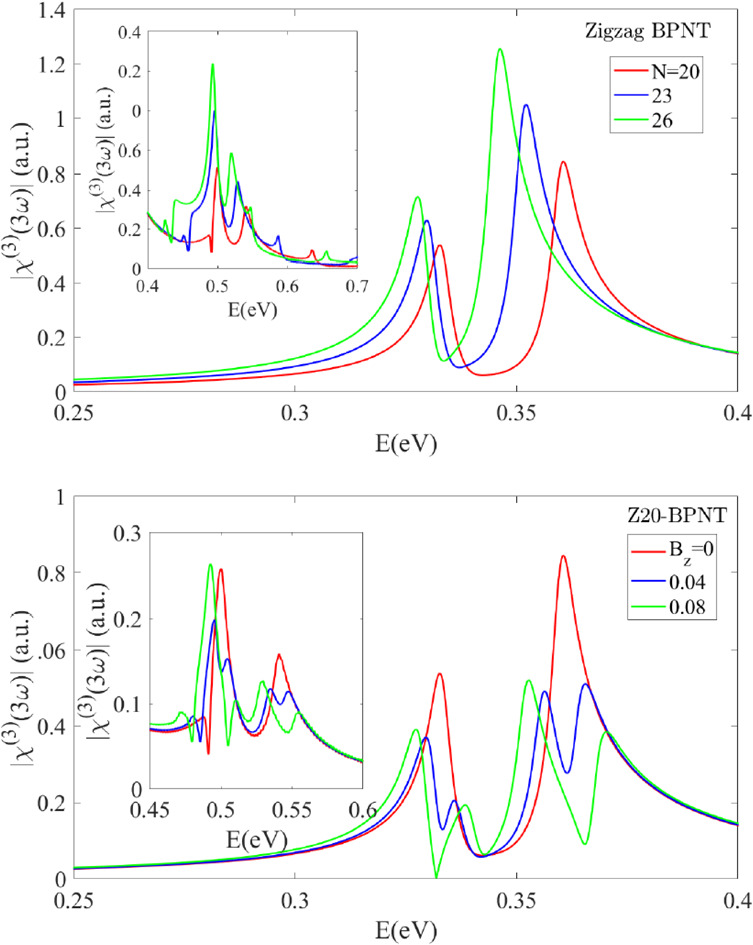

## 1 Introduction

The reduction of physical systems to the nanoscale has led to the emergence of a new class of materials with remarkable electronic, optical, and mechanical properties that have initiated profound developments in various fields of technology.^1^ Owing to their exceptional mechanical strength and remarkable electrical and thermal conductivity, these materials have unlocked new opportunities in quantum-scale material engineering.^2,3^ Furthermore, they are paving the way for next-generation technologies with wide-ranging applications in sensors, electronic devices, and energy storage systems.

Among nanoscale structures, carbon nanotubes (CNTs) represent one of the most prominent examples. They are formed by rolling a single graphene sheet into a cylindrical geometry, uniquely characterized by a chiral vector (*n*, *m*) that determines their chirality, diameter, and symmetry.^4,5^ This geometrical dependence directly governs their exceptional electronic properties, allowing CNTs to behave as either metals or semiconductors.^6^ Such sensitivity to structure gives rise to a wide spectrum of electronic and optical responses, making CNTs as promising candidates for nanoscale electronic applications such as transistors.^7^ Their electronic behavior can be further tailored through doping, mechanical strain, or external electric and magnetic fields.^7–11^ For instance, a transverse electric field induces Stark shifts that modify subband mixing and band gaps, while an axial magnetic field generates the Aharonov–Bohm effect, periodically modulating the gap.^12–14^ These mechanisms provide dynamic control over CNT properties, which is essential for the design of advanced optoelectronic devices.

The remarkable properties of CNTs have motivated researchers to explore analogous nanotubular structures composed of different two-dimensional (2D) sheets, particularly III–V materials such as boron nitride (BN) and boron phosphide (BP).^15,16^ Unlike CNTs, which can exhibit either metallic or semiconducting behavior, these nanotubes are inherently semiconducting, due to the electronegativity difference between their constituent atoms.^17^ Similar to CNTs, their electronic properties can be tailored through mechanical strain, external fields, doping, or molecular adsorption, enabling the development of multifunctional materials with tunable characteristics.^18–20^ Among them, BN nanotubes (BNNTs) have been extensively investigated due to their wide band gap and exceptional chemical stability, while BP nanotubes (BPNTs) exhibit a moderate band gap that makes them particularly promising for optoelectronic applications in the visible and near-infrared regimes.^21–23^

In their two-dimensional form, monolayer and bilayer hexagonal boron phosphide (h-BP) exhibit distinctive structural, electronic, and optical characteristics. The monolayer is a direct-gap semiconductor with a band gap of ∼0.9 eV, which decreases in the bilayer due to interlayer coupling.^23^ It also features low carrier effective masses, leading to high carrier mobility and superior mechanical stability compared to many other 2D materials.^24^ Its electronic structure is highly tunable through doping, enabling the realization of both p-type and n-type semiconductors.^24–26^ External perturbations such as electric fields can further modulate the band gap, even inducing a transition from direct to indirect.^27,28^ Similarly, tensile or compressive strain can tune the band gap and enhance optical responses such as light absorption.^29,30^ While the optical properties remain isotropic under biaxial strain, uniaxial strain introduces strong anisotropy.^31^

Rolling up a BP sheet into a BPNT introduces curvature-induced hybridization and symmetry breaking while retaining the intrinsic electronegativity difference of BP, resulting in properties distinct from CNTs. Unlike CNTs, BPNTs consistently display semiconducting behavior with reduced sensitivity to chirality. *Ab initio* studies confirm the dynamical and thermodynamic stability of BPNTs across a broad range of diameters and chiralities. Their diameter increases linearly with the chiral index, while the chemical potential and electrophilicity index of zigzag BPNTs exhibit clear chirality-dependent variations.^32^ Furthermore, theoretical studies indicate that zigzag BPNTs are soluble in polar solvents, enabling their use as carriers for bio-relevant molecules such as nedaplatin, thereby opening avenues for biomedical applications.^24^

The electronic properties of BPNTs can be effectively engineered through substitutional doping, molecular adsorption, or external perturbations. For instance, carbon doping narrows the band gap,^33,34^ while adsorption of H_2_O_2_ slightly reduces the gap and enhances conductivity.^35^ Lithium adsorption can even induce a semiconductor-to-metal transition,^36^ making BPNTs attractive for hydrogen storage and battery technologies.^37^ Beyond energy storage, BPNTs have been proposed as efficient catalysts for CO conversion into economically viable products.^38^ Doping with transition metals (*e.g.*, Co) further enhances their potential in drug delivery, as demonstrated for metformin transport.^39^ Additionally, BPNTs exhibit high stability and their thermal properties, such as thermal conductivity, are improved by doping or external fields, making them suitable for thermoelectric applications.^40^

Compared with BNNTs, which exhibit wide band gaps and deep-UV activity, BPNTs offer a narrower and more versatile band gap suitable for visible to near-infrared applications. Their absorption spectra show strong chirality dependence, with optical peaks undergoing red- or blue-shifts as the tube diameter increases.^41^ This tunability makes BPNTs promising candidates for optoelectronic devices requiring controlled spectral responses.

Despite these significant findings on the electrical, optical, and thermodynamic properties, systematic studies of the optical response of BPNTs remain limited, particularly with respect to nonlinear optical (NLO) effects such as higher harmonic generation. Addressing this gap is crucial, as NLO properties are essential for developing the next generation of optical devices, including switches, limiters, and frequency converters. Moreover, the influence of external magnetic fields, caused by Aharonov–Bohm phase modulation, provides an additional degree of tunability, opening prospects for field-controlled one-dimensional photonic devices.

The present study aims to investigate the linear and nonlinear optical properties of BPNTs, with a particular focus on third-harmonic generation (THG). We employ the fifth nearest-neighbor tight-binding model combined with the density-matrix formalism, a framework successfully applied to CNTs, to systematically analyze the frequency-dependent third-order susceptibility of BPNTs and the effects of axial magnetic fields on their optical spectra. To our knowledge, this work provides the first comprehensive account of nonlinear optics in BPNTs and establishes their potential for the design of tunable nanophotonic and quantum devices.

## 2 Computational approach

### 2.1 Tight-binding Hamiltonian for monolayer h-BP

The electronic band structure of monolayer h-BP is obtained within a tight-binding model, which is well suited for capturing the electronic and optical properties of 2D materials and their nanostructures. The primitive unit cell of pure h-BP contains two nonequivalent atomic species (B and P). In the basis of |*ϕ*_B_ and |*ϕ*_P_, the Bloch Hamiltonian is expressed as a 2 × 2 matrix:1
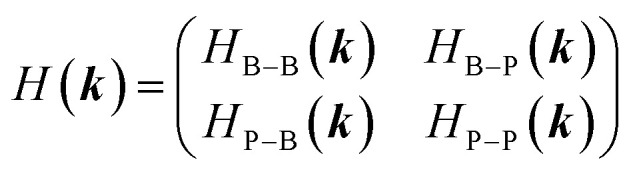


The wave vector ***k*** = (*k*_*x*_, *k*_*y*_) is defined within the reciprocal lattice of monolayer h-BP structure within the first Brillouin zone. Diagonal elements include on-site energies Δ_X_ (X ∈ {B, P}) and same-sublattice hoppings (here up to second [*t*_2_] and fifth [*t*_5_] nearest neighbors), while off-diagonal elements include B–P hoppings (here up to first [*t*_1_], third [*t*_3_] and fourth [*t*_4_] nearest neighbors). Writing **R**_*m*_ for the *m*-th neighbor vectors and *t*_XY_^[*m*]^ for the corresponding hopping amplitudes, the Hamiltonian matrix elements can be written as:2a
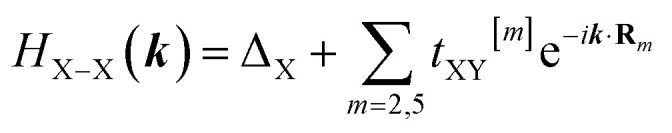
2b
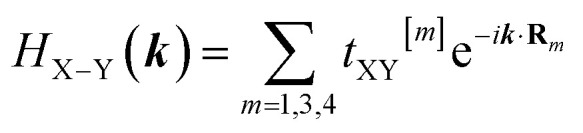


The hopping parameters *t*_XY_^[*m*]^ and on-site energies Δ_X_ used in this work were determined by fitting the tight-binding band structure to density functional theory (DFT) calculations. This ensures that our model accurately reproduces the essential electronic features of the h-BP structure. The optimized parameters are summarized in Table 1.

**Table 1:** The 5-th nearest-neighbor tight-binding hopping parameters for BPNTs

*t*_1_	*t*_2_	*t*_3_	*t*_4_	*t*_5_
1.8511	0.2111	0.2095	0.0433	0.0807

The band structure follows from3
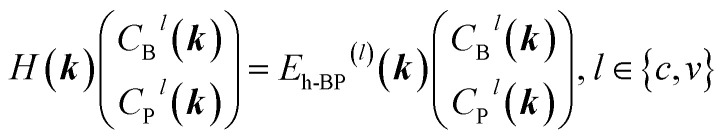
with eigenvalues4
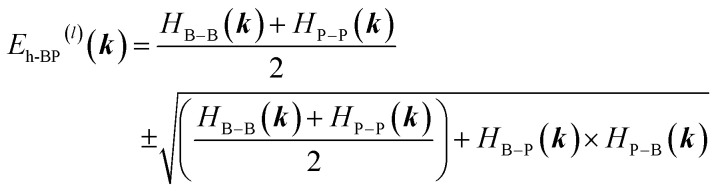
where ± correspond to conduction and valence bands, respectively. Fig. A1 (SI) compares the 5NN-TB bands (dotted red) and DFT bands (solid green) along the standard high-symmetry path, showing good agreement and a direct band gap at the *K* point.

### 2.2 Electronic structure of boron–phosphide nanotubes (BPNTs)

Within the zone-folding approximation,^6,42^ the electronic structure of a BPNT is generated by quantizing the continuous wavevector ***k*** = (*k*_*x*_, *k*_*y*_) of h-BP around the tube circumference while retaining a continuous component along the tube axis. Let 
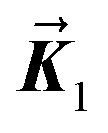
 and 
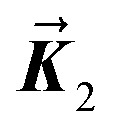
 be reciprocal vectors associated with the circumferential and axial directions, respectively. Then the wave vector of the BPNTs can be given by:^43,44^5
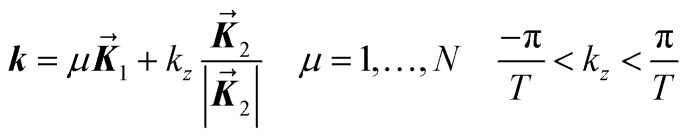
where *T* is the translational period along the tube axis and *N* is the number of hexagons in the nanotube unit cell. Using eqn (4) and (5), the BPNT subband energies are given by:6
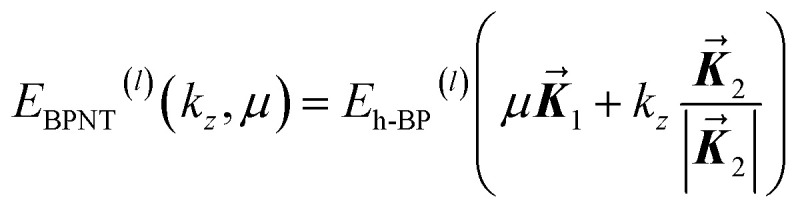
When an axial magnetic field *B*_0_ is applied parallel to the nanotube axis, it induces an Aharonov–Bohm phase shift in the electron wavefunction.^45–47^ This phenomenon modifies the hopping parameters *via* the Peierls substitution:^48^7
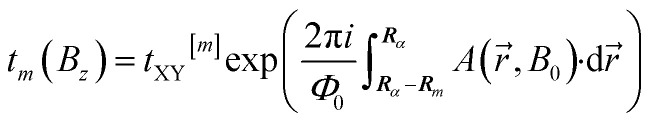
where 
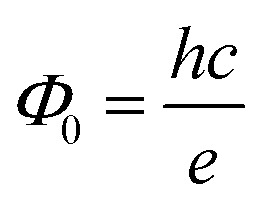
 is the magnetic quantum flux and 
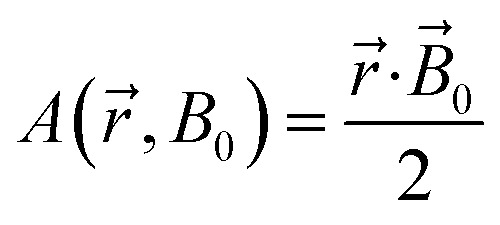
 is the magnetic vector potential in the symmetric gauge. The dimensionless parameter *B*_*z*_ = π*R*_0_^2^*B*_0_/*Φ*_0_ represents the magnetic flux passing through the nanotube's cross-section of radius *R*_0_.^43^ This phase shift effectively alters the periodic boundary condition for the circumferential motion, resulting in a shift of the subband index *E*(*k*_*z*_, *μ*) → *E*(*k*_*z*_, *μ* + *B*_*z*_).^49^ For a zigzag BPNT with a radius of approximately 1 nm and 2 nm, a magnetic flux of *B*_*z*_ = 0.1 corresponds to magnetic field strengths of approximately *B*_0_ = 132 T and 33 T, respectively.

### 2.3 Linear optical properties

To explore the optical properties of BPNTs, the calculation of dipole matrix elements is essential. Using the derived band structure and corresponding coefficients *C*(***k***), the allowed interband transitions between the valence and conduction bands are evaluated through the optical matrix elements, expressed as *D*(***k***) = <*Ψ*^f^_***k***_(***r***)|∇|*Ψ*^i^_***k***_(***r***)>. In this context, the [*Ψ*^i^] and [*Ψ*^f^] are the initial and final wave functions, respectively, and the interband dipole matrix elements are defined as follows:^42,43^8
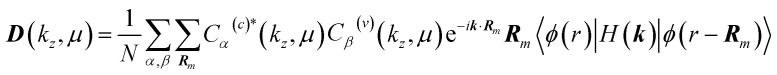
where *ϕ*(***r*** − ***R***_*m*_) exhibits the *p*_*z*_ atomic wave function for atoms and the ***R***_*m*_ denotes the vector from an origin atom to its *m*-th neighbor atoms. For polarization parallel to the nanotube axis, the allowed transitions occur between valence and conduction subbands with the same *μ* and *k*_*z*_ indexes.^50^ The ***k*** dependence of electric dipole moment *d*(***k***) corresponding to transitions between the valence and conduction bands with an energy difference *E*^*cv*^(***k***) is related to the dipole matrix elements by 
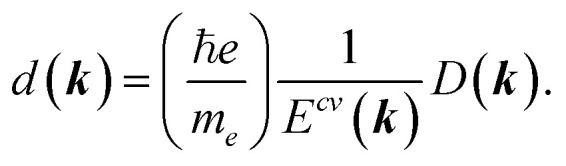


In this study, the linear optical properties of the BPNTs are investigated using the density matrix formalism combined with perturbation theory. The time evolution of the density matrix, *ρ*(*t*), is given by:^44^9
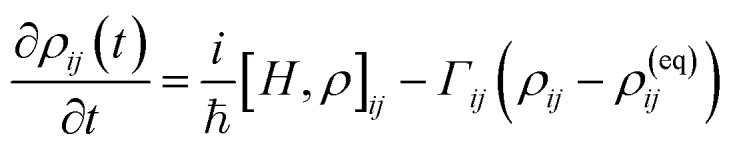
Here, the total Hamiltonian is *H* = *H*_0_ + *V*_int_, where *H*_0_ is the unperturbed Hamiltonian of the system and 
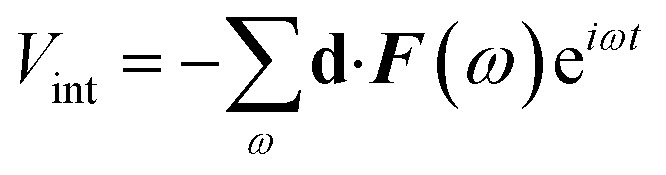
 represents the light–matter interaction in the electric dipole approximation. **d** = *e****r*** is the electric dipole moment operator and ***F*** is the electric field of the incident electromagnetic wave. Furthermore, *ρ*^(eq)^_*ij*_ is the density matrix at thermal equilibrium, and *Γ*_*ij*_ is a phenomenological decay rate.

By applying perturbation theory, the *v*-th order contribution to the density matrix, *ρ*_*ij*_^(*v*)^(*t*) can be obtained 
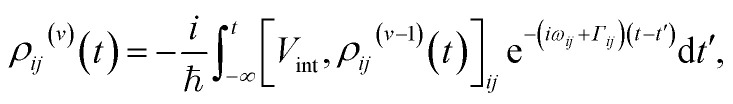
 with *ℏω*_*ij*_ = *E*_*j*_ − *E*_*i*_ as the energy difference between the eigenstates *i* and j. Considering the allowed optical transitions between the valence [denoted as *i* → (*v*, *k*)] and conduction [denoted as *ij* → (*c*, *k*′)] bands, the optical susceptibility *χ*^(1)^(*ω*) is derived as follows:^51^10
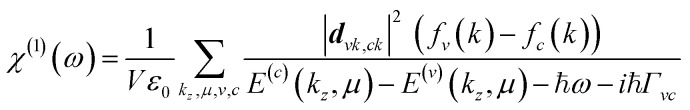


The dipole matrix element, *d*_*vk*,*ck*′_ represents optically allowed transitions between a valence band state at (*v*, *k*) and a conduction band state at (*c*, *k*′). Its magnitude directly determines the intensity of the associated optical peak. The term *ℏΓ*_*vc*_ ≡ *ℏΓ*_0_, derived from a semiclassical description of the density matrix evolution, represents dephasing and scattering effects within the optical response of the material and in realistic systems leads to spectral broadening of the optical features. In the present calculations, a phenomenological broadening parameter of 50 meV is employed.

Furthermore, the quadratic electro-optic (QEO) effect can be related to the linear susceptibility *via* the following relation:^52^11
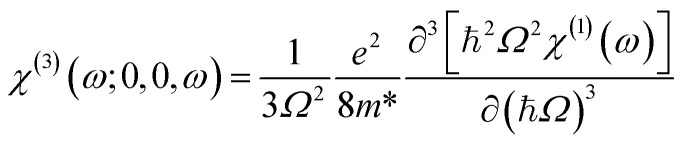
where the complex frequency is defined as *Ω* = *ω* + *iΓ*_0_.

### 2.4 Third-order nonlinear optical susceptibility

The third-order nonlinear polarization, *P*^(3)^_*α*_(*Ω*), at a frequency *Ω* is generally expressed in terms of the third-order susceptibility tensor, 
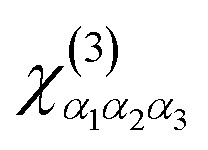
:^51^12
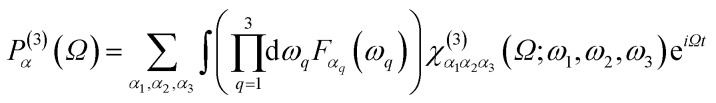


The calculation of this tensor can be efficiently performed using the Genkin–Mednis formalism,^53^ which provides a compact expression for *χ*^(3)^.^54^

Within this framework, *χ*^(3)^(*Ω*; *ω*_1_, *ω*_2_, *ω*_3_) is separated into interband and mixed inter/intraband contributions:

13 *χ*^(3)^(*Ω*; *ω*_1_, *ω*_2_, *ω*_3_) = *χ*^(3)^_inter_(*Ω*; *ω*_1_, *ω*_2_, *ω*_3_) + *χ*^(3)^_mix_(*Ω*; *ω*_1_, *ω*_2_, *ω*_3_)

For allowed optical transitions between the valence [(*v*, *k*)] and conduction [(*c*, *k*′)] states, the two components for the selected BPNTs are:^54–56^14a
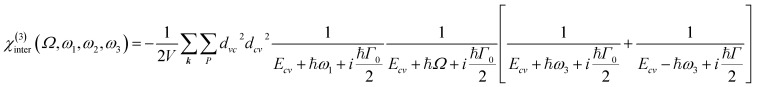
14b
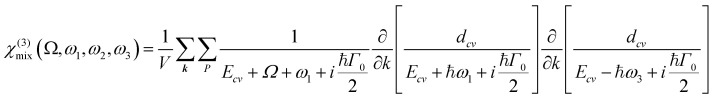


The summation over *P*, which results in 24 terms, includes all possible permutations of the incident photon frequencies *ω*_*j*_ and *Ω* = −(*ω*_1_ + *ω*_2_ + *ω*_3_), ensuring full symmetry of the third-order nonlinear susceptibility. The intensity-dependent index of refraction and the two-photon absorption spectra of the selected BPNTs can be obtained from eqn (14) as:

15 *χ*^(3)^_IDIR_(*ω*) ≡ *χ*^(3)^(*Ω* = −*ω*, *ω*_1_ = *ω*, *ω*_2_ = *ω*, *ω*_3_ = −*ω*)

where the frequency set (*Ω*, *ω*_1_, *ω*_2_, *ω*_3_) is completed by including all possible permutations of the incident photon frequencies *ω*_*j*_.

## 3 Results and discussion

To the best of our knowledge, this work presents the first theoretical investigation into the linear and nonlinear optical properties of BPNTs. Utilizing a comprehensive tight-binding model, we explore how their electronic and optical characteristics can be modulated by fundamental parameters including the nanotube radius (related to the chiral index *n*) and an external axial magnetic field Bz, focusing specifically on the zigzag BPNTs with 20 ≤ *n* ≤ 28.

### 3.1. Linear optical response: the role of nanotube radius

**Fig. 1:**
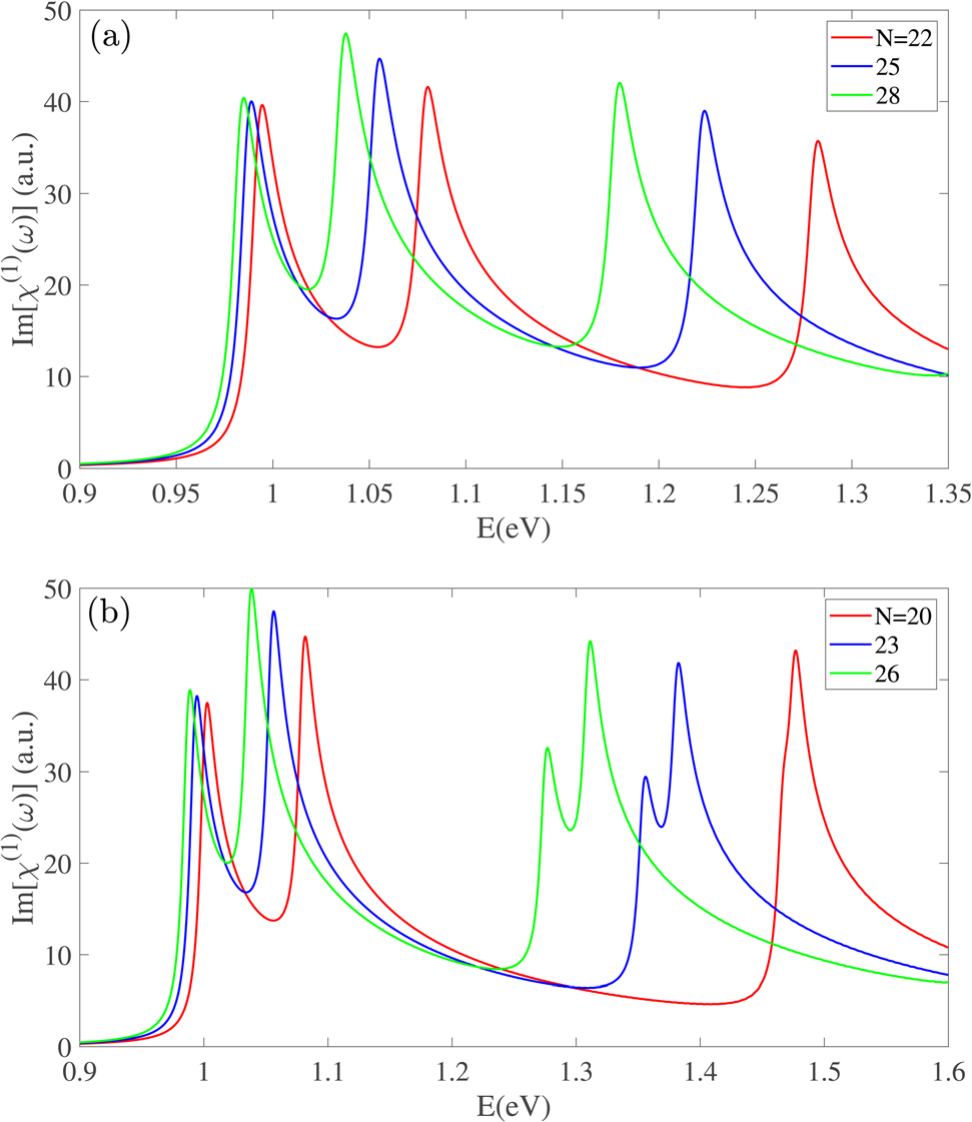
The imaginary part of the linear optical susceptibility for two families of zigzag BPNTs (a) S_1_ and (b) S_2_. The spectra show a systematic red-shift of the primary infrared absorption peaks as the nanotube radius increases.

The optical response of a material to an external electromagnetic field is fundamentally described by its optical susceptibility. Fig. 1 displays the imaginary part of the linear optical susceptibility, Im[*χ*^(1)^(*ω*)], for selected zigzag (*n*, 0) BPNTs (Zn-BPNTs) from two distinct structural families, S_1_ [with *n* = 3*s*_0_ + 1] and S_2_ [with *n* = 3*s*_0_ + 2], within the infrared energy range (*E* < 2 eV). This classification, based on *n* mod 3 and originating from the tight-binding and zone-folding formalism widely applied to carbon, Si, Ge, and BN nanotubes,^6,43,57^ enables us to systematically capture radius-dependent trends such as the red-shift of optical peaks and the emergence of regular family-dependent spectral patterns. For completeness, additional results for the S_3_ (*n* = 3*s*_0_) family are provided in the SI.

The spectral peaks in Im[*χ*^(1)^(*ω*)] correspond to allowed optical transitions between the *i*-th valence sub-band [*E*_*i*_^(*v*)^(*k*)] and the *j*-th conduction sub-band [*E*_*j*_^(*c*)^(*k*)]. These transitions are governed by dipole selection rules, which for nanotubes, only allow vertical transitions where *i* = *j* and *k* = *k*′.^42^ The energy of each peak is directly related to the energy difference between these valence and conduction subbands.

In the absence of external perturbations, each Zn-BPNT exhibits 2*n* valence and 2*n* conduction subbands with twofold degeneracy for states indexed by *m* and (2*n* − *m*). Consequently, transitions originating from these degenerate pair subbands, namely Δ*E*_*m*_^(*cv*)^ ≡ *E*_*m*_^(*v*)^ → *E*_*m*_^(*c*)^ and Δ*E*_(2*n*−*m*)_^(*cv*)^ ≡ *E*_*m*_^(*v*)^ → *E*_*m*_^(*c*)^, contribute to the same optical peak, enhancing its intensity. For instance, the first prominent peak for the Z20-BPNT arises from the degenerate transitions Δ*E*_13_^(*cv*)^ and Δ*E*_27_^(*cv*)^, while for the Z22-BPNT, it stems from the Δ*E*_15_^(*cv*)^ and Δ*E*_29_^(*cv*)^ transitions.

A notable feature across all studied Zn-BPNTs is the absence of any significant optical absorption peaks for energies below ∼0.95 eV. This is a direct consequence of the intrinsic electronic band gap centered at the *Γ* point of the Brillouin zone, which originates from the differing electronegativity of B and P atoms in the primitive unit cell. Beyond the fundamental band gap region, the optical spectra of BPNTs within each family display a systematic evolution as the index *n* increases. An increase in radius leads to a reduction in the energy separation between adjacent subbands in both the valence and conduction bands. This modification of the electronic structure directly impacts the transition energies (Δ*E*_*m*_^(*cv*)^) causing a distinct red-shift of the optical peaks in the infrared range with increasing *n*. This phenomenon is most pronounced for the first optical peak in Fig. 1, which corresponds to the fundamental band gap transition, indicating a strong tunability of the band gap with the nanotube radius. This red-shift is also observed for subsequent peaks at higher energy ranges, accompanied by a reduction in the energy spacing between adjacent peaks, leading to a denser optical spectrum for larger-radius BPNTs. As shown in Fig. 1(a), S_1_ category nanotubes characteristically display a greater number of peaks within a smaller energy window compared to their S_2_ counterparts. Furthermore, for large *n* values, S_2_ BPNTs exhibit the emergence of dual-peak features, originating from transitions between nearly-degenerate subbands [Fig. 1(b)]. This geometric tunability of optical properties is one of the outstanding features of low-dimensional systems and highlights the potential for engineering the optical response of BPNTs by precise structural control.

**Fig. 2:**
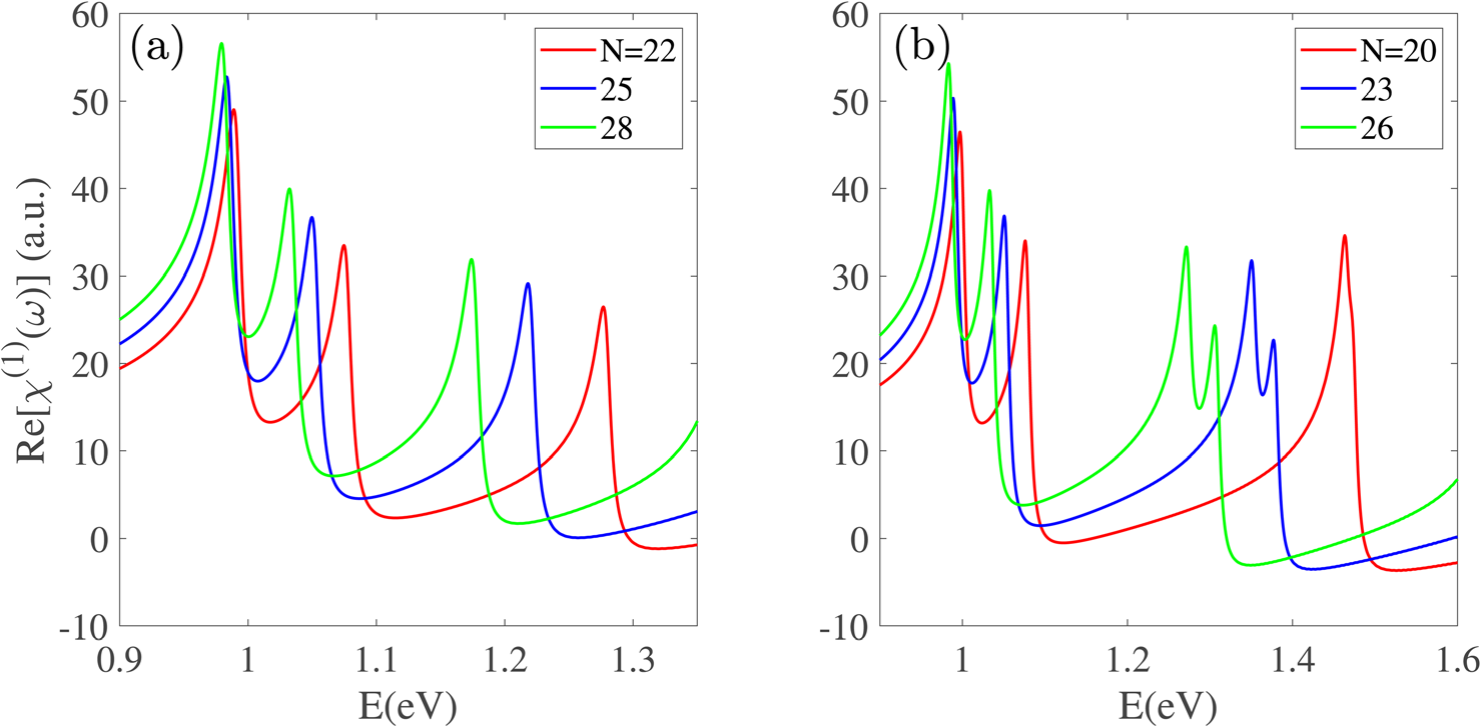
The real part of the linear optical susceptibility for (a) S_1_ and (b) S_2_ nanotube types. The static dielectric constant (*ω* → 0) is shown to increase with the nanotube radius and the spectral peaks undergo the radius-dependent red-shifts.

Fig. 2 presents the real part of the linear susceptibility, Re[*χ*^(1)^(*ω*)], which complements the findings from the imaginary part. The static dielectric constant, Re[*χ*^(1)^(*ω* → 0)], is observed to increase with the nanotube radius. The peak positions in Re[*χ*^(1)^(*ω*)] as well as their red-shift with increasing *n* show a perfect agreement with the behavior of Im[*χ*^(1)^(*ω*)].

**Fig. 3:**
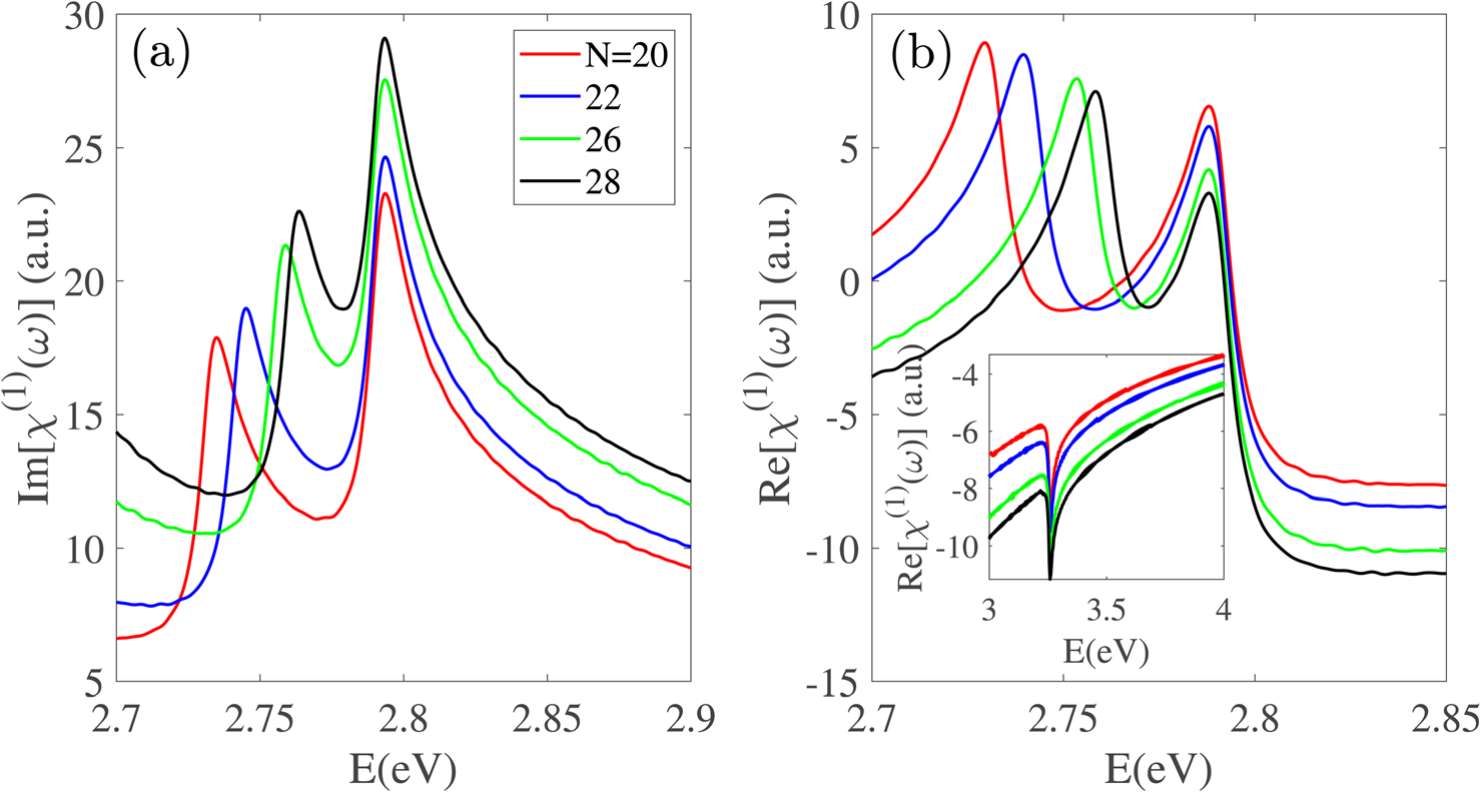
The (a) imaginary and (b) real part of the linear optical response of even-index BPNTs in the visible energy range. With an increasing nanotube radius, the first peak exhibits a blue-shift, while a prominent peak at approximately 2.8 eV remains at a constant position but increases in intensity.

In the visible energy range (2.5 < *E* < 3.0 eV), the optical response of Zn-BPNTs with even indices *n* follows a distinct and uniform pattern, as depicted in Fig. 3. These nanotubes consistently show two prominent peaks. As the radius increases, the first peak exhibits a blue-shift, while the energy separation between the two peaks decreases. The magnitude of this blue-shift demonstrates a linear dependence on the index *n*. Intriguingly, all even-index Zn-BPNTs display a persistent peak at approximately 2.8 eV whose position remains unchanged with an increasing the nanotube radius, although its intensity increases. The physical origin of this constant peak is attributed to optical transitions between specific valence and conduction subbands whose energy separation is largely insensitive to changes in radius. The increasing intensity is primarily due to the enhancement of dipole matrix elements for these transitions, in larger-radius nanotubes. The real part of the susceptibility, Re[*χ*^(1)^(*ω*)], shown in Fig. 3(b), reflects these trends, displaying a corresponding blue-shift for the first peak and an increasing intensity for the constant peak at 2.8 eV. Additionally, a peak with negative intensity appears around 3.2 eV, which also remains constant in energy, but its magnitude increases as the nanotube radius increases.

### 3.2. Nonlinear optical response: the effect of nanotube radius

Regarding the dependence of the linear optical spectrum on the index *n*, similar effects are observed in the nonlinear optical spectra. These arise from the modifications in the electronic band structure of Zn-BPNTs and its strong dependence on the chiral index *n*, leading to pronounced changes such as noticeable shifts of optical peaks across different energy regions.

**Fig. 4:**
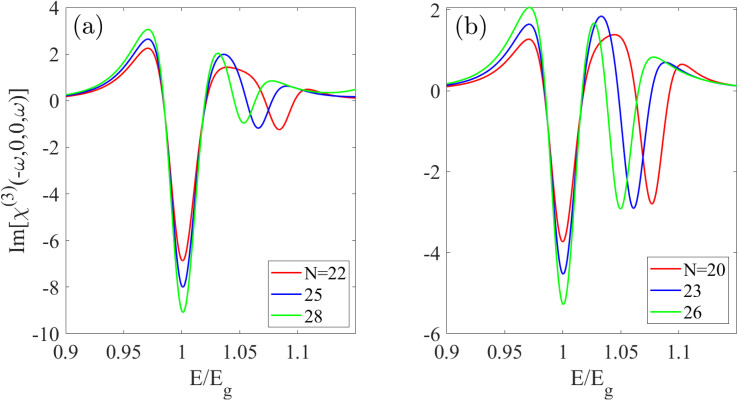
The quadratic electro-optic (DC Kerr) effect for (a) S_1_ and (b) S_2_ BPNTs. The spectra show that as the nanotube radius increases, peak intensities grow for energies below the band gap, while peaks at *E* > *E*_g_ exhibit a noticeable red-shift.

Fig. 4 illustrates the quadratic electro-optic (DC Kerr) effect *χ*_DC_(*ω*) ≡ *χ*^(3)^(−*ω*, 0, 0, *ω*), for S_1_ and S_2_ Zn-BPNTs as a function of scaled energy (*E*/*E*_g_). Far from the first optical resonance peak, the response is negligible due to the non-zero band gap. However, near resonant frequencies, the spectrum is characterized by multiple peaks of both positive and negative sign. For energies *E* ≤ *E*_g_, the peak intensities increase with *n*, while their positions on the scaled energy axis remain nearly constant. In contrast, for energies *E* > *E*_g_, the peaks exhibit a noticeable red-shift relative to *E*/*E*_g_ with increasing *n*, accompanied by a negligible decrease in intensity. This behavioral pattern is consistent for both S_1_ and S_2_ nanotube types in the visible energy range.

**Fig. 5:**
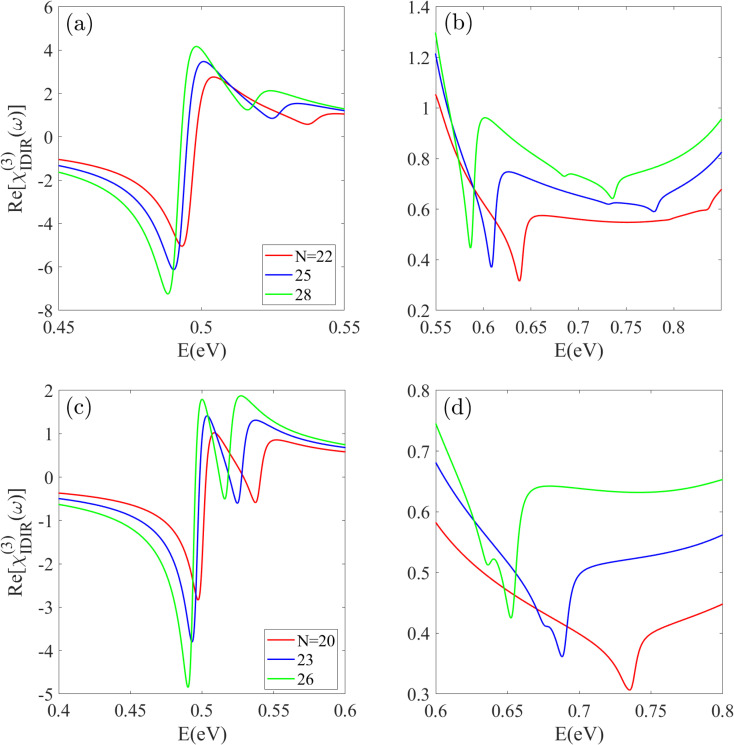
The *χ*^(3)^_IDIR_(*ω*) spectrum for (a and b) S_1_ and (c and d) S_2_ nanotube types. A strong negative-intensity peak emerges near *E*_g_/2, corresponding to a two-photon transition, which exhibits a red-shift with increasing radius.

The real part of the *χ*^(3)^_IDIR_(*ω*), is shown in Fig. 5 for the infrared region. This spectrum corresponds to the intensity-dependent nonlinear refractive index, also known as the Optical Kerr Effect. The features in this spectrum are driven by multi-photon resonances, primarily two-photon absorption (TPA) processes. As the energy approaches half the band gap, a prominent negative peak appears near ≈*E*_g_/2, corresponding to a two-photon transition between the highest valence subband and the lowest conduction subband at the *Γ* point. The position of this primary TPA peak exhibits a red-shift with increasing *n*. For the S_1_ group, it shifts from 0.49 eV for Z22-BPNT to 0.48 eV for Z28-BPNT [Fig. 5(a)]. The shift is more pronounced for the S_2_ group, moving by approximately 0.13 eV as the radius increases from *n* = 20 to *n* = 26. At higher energies beyond *E*_g_/2, additional peaks appear. For S_1_ nanotubes, shoulder peaks around *E* ≈ 0.5 eV and significant negative peaks (*e.g.*, at 0.537 eV and 0.516 eV for Z22 and Z28-BPNTs, respectively) are observed, which arise from transition between the second valence and conduction subbands near the Fermi level at the *Γ* point.

A similar behavior is observed for the S_2_ nanotubes, where both the primary and secondary peaks exhibit a clear red-shift as the index *n* increases. Specifically, Z20 to Z26-BPNTs show two distinct negative-intensity peaks: the first appearing near 0.50 eV and the second within the 0.51–0.53 eV range. The positions of these peaks gradually shift to lower energies with increasing nanotube radius, reflecting the strong *n*-dependence of the two-photon absorption response. Just below the band gap energy, additional peaks are resolved, corresponding to transitions involving the third-nearest valence and conduction subbands to the Fermi level. These peaks have significantly lower magnitudes [several orders smaller] than the first and second peaks. As illustrated in Fig. 5(b) and (d), they appear within the 0.58–0.63 eV range for S_1_ nanotubes and 0.65–0.73 eV for S_2_ nanotubes. Notably, S_2_ nanotubes with *n* > 23 display negative dual-peaks, a feature attributed to the small energy separation between the relevant subbands in these larger-radius structures.

**Fig. 6:**
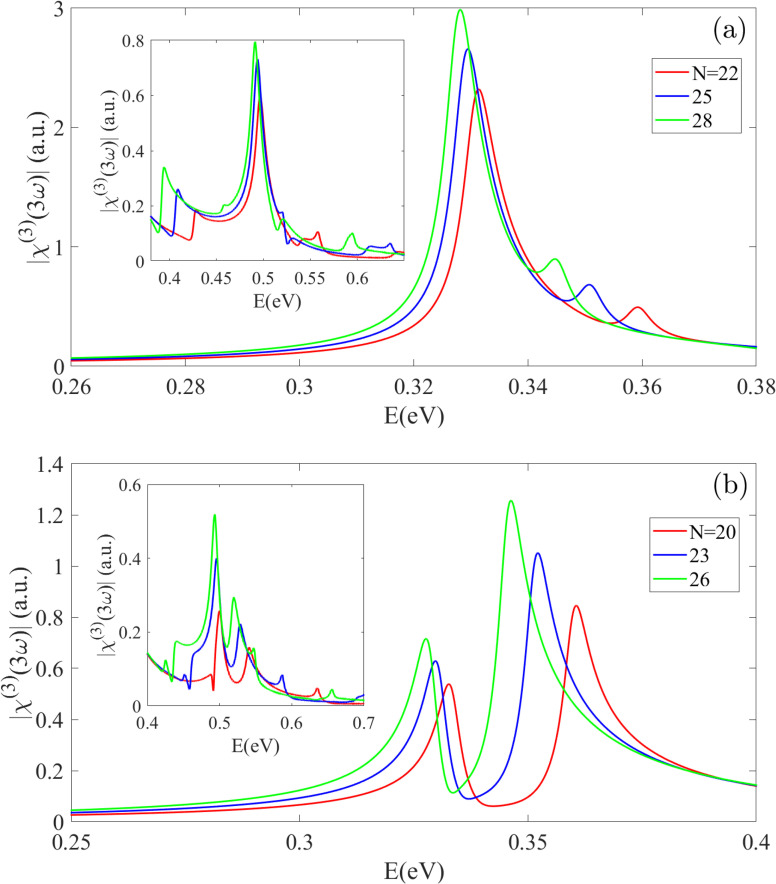
The third-harmonic generation spectrum for (a) S_1_ and (b) S_2_ nanotube types. The dominant THG peak occurs at approximately *E*_g_/3, with its intensity increasing and exhibiting a red-shift as the nanotube radius grows. Unlike CNTs, BPNTs show multi-peak THG features, highlighting their unique nonlinear response arising from their intrinsic semiconducting character.

Fig. 6 presents the third-harmonic generation (THG) spectrum, *χ*^(3)^_THG_(3*ω*) for S_1_ and S_2_ BPNTs over various energy ranges, which arises from three-photon absorption processes, due to the allowed dipole selection rules. For Z22-BPNT (from S_1_ type), a dominant peak is observed at approximately *E* = 0.33 eV ≈ *E*_g_/3, resulting from a three-photon resonance between the nearest valence and conduction subbands to the Fermi level at the *Γ* point. The existence of such a strong peak is consistent with previous studies on CNTs using single-band model and DFT methods.^54,55,58^ However, our comprehensive model also predicts a second peak with significantly weaker intensity at 0.36 eV in Z22-BPNT, arising from a three-photon transition between the second-nearest subbands relative to the Fermi level. This feature is not typically observed in CNTs and is a direct consequence of the unique, inherently semiconducting nature of BPNTs. A key advantage of our tight-binding model, which incorporates all allowed transitions across the entire Brillouin zone, is its ability to predict higher-energy THG peaks beyond the first peak region, missed by simplified models.^55^ As shown in the insets of Fig. 6, BPNTs exhibit distinct THG peaks at energies approaching the band gap, though their intensity diminishes. For example, S_1_ nanotubes exhibit several peaks at a nearly constant energy of 0.5 eV, while S_2_ nanotubes display dual peaks in the same region, all of which increase in intensity with the nanotube radius. With increasing radius, the THG peaks in the infrared region undergo a red-shift and an increase in intensity. In Z28-BPNT, the first two peaks shift to 0.328 eV and 0.345 eV, respectively. S_2_ nanotubes show notable dual peaks below 0.4 eV, where, contrary to S_1_, the second peak is more intense than the first. These peaks also red-shift with increasing *n* (*e.g.*, from 0.333 and 0.36 eV for Z20-BPNT to 0.327 and 0.346 eV for Z26-BPNT), accompanied by an intensity increase.

### 3.3. Magneto-optical response: the effect of an axial magnetic field

An axial magnetic field (*B*_z_) provides a powerful means to modulate the electronic structure and consequently, the optical properties of BPNTs. The magnetic field induces an Aharonov–Bohm phase, which breaks the time-reversal symmetry of the system. This lifts the twofold degeneracy of the *m* subbands, causing them to split in energy.^56^ This phenomenon, previously reported for CNTs, BNNTs, and SiNTs,^12,59^ strongly alters the optical spectra of BPNTs.

**Fig. 7:**
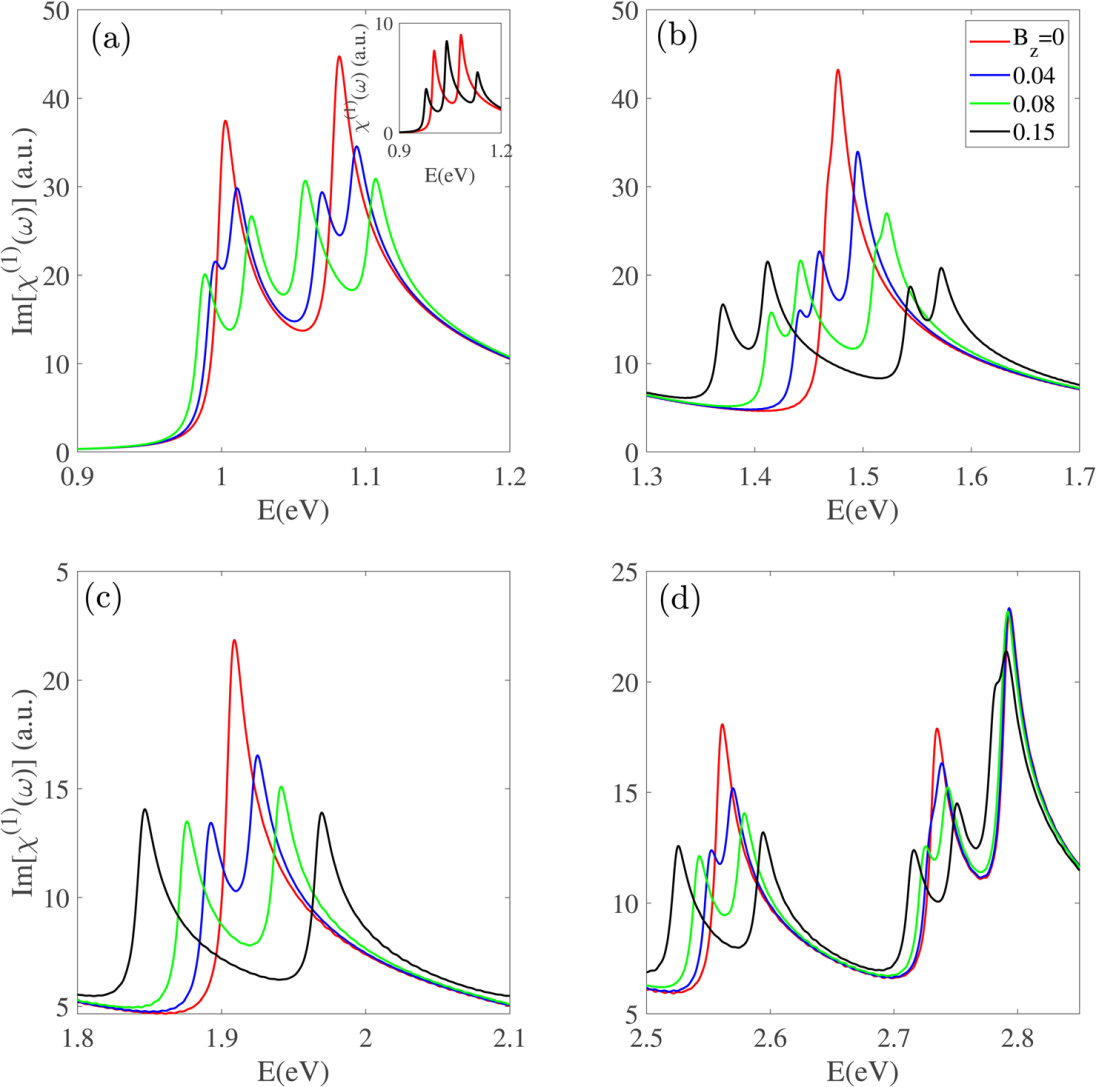
(a)–(d) The effect of an axial magnetic field (Bz) on the linear susceptibility of a Z20-BPNT. The application of a magnetic field lifts subband degeneracy, leading to the splitting of each original optical peak into two components, one red-shifted and one blue-shifted.

Fig. 7 shows the effect of *B*_z_ on the Im[*χ*^(1)^(*ω*)] spectrum of Z20-BPNT across different energy ranges. In the absence of a field, this nanotube has two prominent peaks at 1.0 eV and 1.1 eV. Applying *B*_z_ lifts the underlying subband degeneracies, causing each original peak (*E*_*ii*_) to split into two new peaks: a red-shifted component (*E*_*ii*_^(–)^) and a blue-shifted component (*E*_*ii*_^(+)^). The energy separation between these split peaks increases linearly with the magnetic field strength. Interestingly, the inset of Fig. 7(a) shows that as the magnetic field increases, the blue-shifted component of the first peak (*E*_11_^(+)^) and the red-shifted component of the second peak (*E*_22_^(+)^) converge, merging into a single high-intensity peak at 1.05 eV under *B*_z_ = 0.15. This feature also occurs at higher energy resonances, where *B*_z_ causes peak splitting and enhances the spacing between neighboring peaks.

The influence of the magnetic field is not uniform across all energy ranges. While most resonance peaks undergo splitting even under weak magnetic fields, certain high-energy features remain largely unaffected. For instance, the prominent peak at ∼2.8 eV shows negligible changes at low magnetic field strengths but clearly splits under a stronger field (*B*_z_ = 0.05). This behavior demonstrates a non-homogeneous magneto-optical response in BPNTs, where high-energy optical transitions demonstrate reduced sensitivity compared to those in the infrared region.

**Fig. 8:**
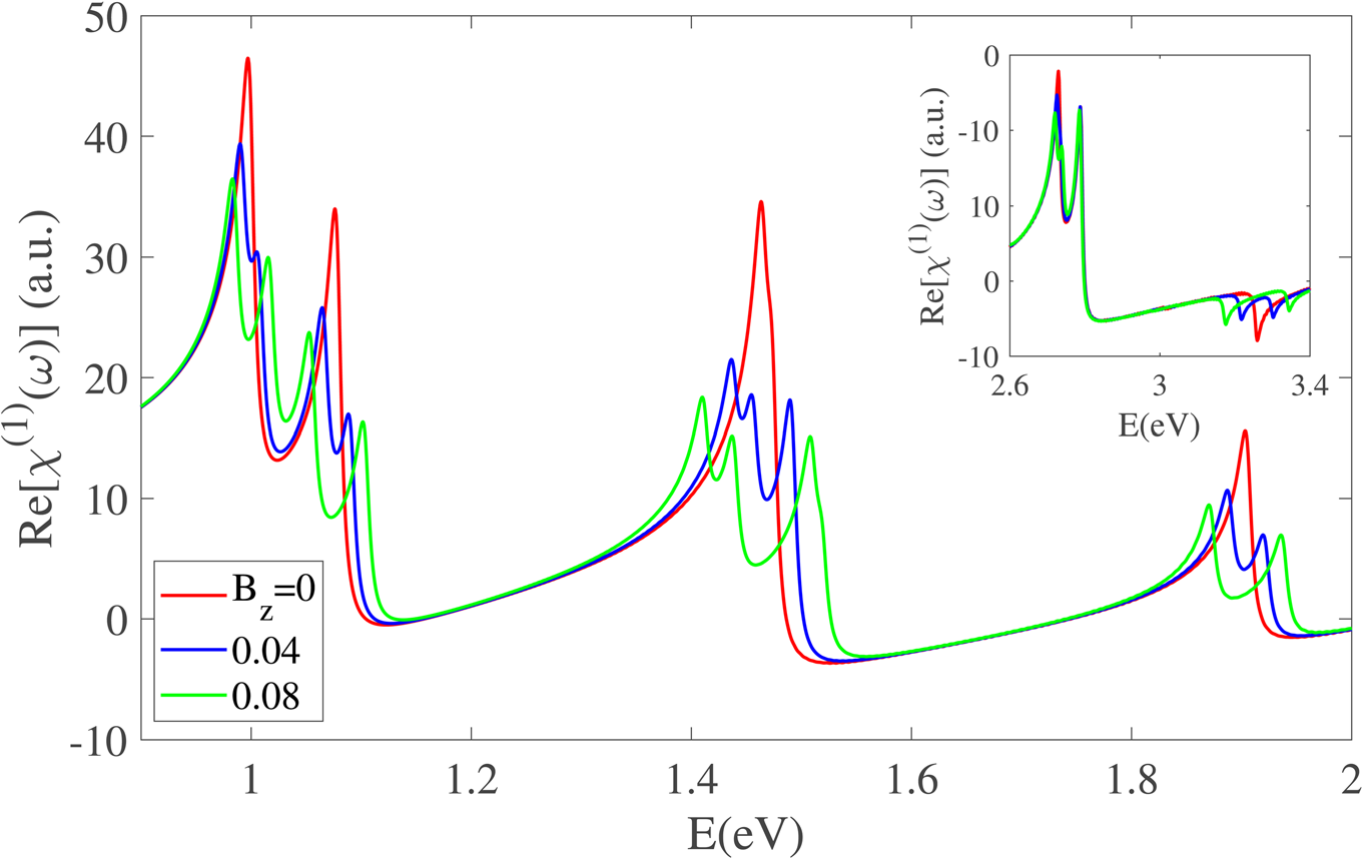
The real part of the linear susceptibility under an axial magnetic field. The results highlight pronounced splitting of peaks across both the infrared and visible ranges.

Consistent with the imaginary part of the linear optical susceptibility, the real part Re[*χ*^(1)^(*ω*)] also exhibits a strong dependence on the applied magnetic field, as shown in Fig. 8 for Z20-BPNT in both the infrared and visible energy ranges. In the infrared regime, all prominent peaks undergo clear splitting under a magnetic field, with the energy separation between the split components increasing as the field strength grows. In the visible region, the spectrum features dual positive peaks around 2.7 eV and a negative peak near 3.25 eV. While the positive peaks are only slightly affected by the magnetic field, the negative peak displays pronounced splitting. These findings indicate that a magnetic field serves as an effective means to induce and tune new resonances across the optical spectrum, with their intensities and positions finely controlled by field strength, an essential property for tailoring BPNTs for advanced optoelectronic applications.

**Fig. 9:**
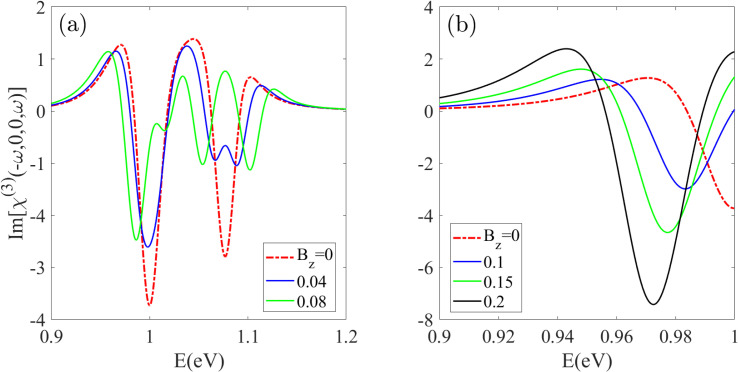
The DC Kerr effect spectrum for a Z20-BPNT under (a) weak and (b) strong magnetic fields. The applied field induces peak splitting, red-shifts, and intensity modifications across the infrared range.

Fig. 9 illustrates the behavior of the Im[*χ*_DC_(*ω*)] spectrum for Z20-BPNT under both weak and strong magnetic fields *B*_z_. Applying a magnetic field *B*_z_ = 0.04 reduces the intensity of the first peak, induces splitting in the second peak and causes noticeable red-shifts in the positive-intensity shoulder features below the first resonant peak [Fig. 9(a)]. A stronger field of *B*_z_ = 0.08 produces splitting across the entire infrared range of the Im[*χ*_DC_(*ω*)] spectrum, indicating that different peaks exhibit varying sensitivity to the magnetic field. As shown in Fig. 9(b), further increasing the field strength results in a significant red-shift and intensity enhancement of the *E*_11_ peak, becoming particularly pronounced at *B*_z_ = 0.2. Moreover, BPNTs with larger radii exhibit more pronounced splitting and red-shifting effects even under relatively weak magnetic fields, highlighting their enhanced magneto-optical tunability.

**Fig. 10:**
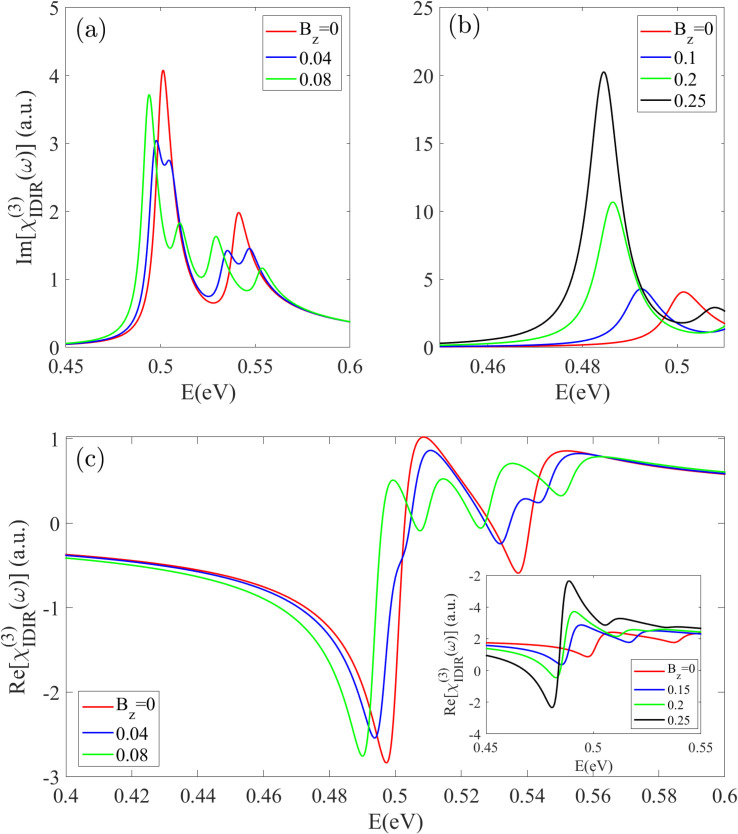
The (a and b) imaginary and (c) real parts of the two-photon absorption spectrum under an increasing magnetic field. The field induces both splitting of the TPA peaks and a substantial red-shift and intensity enhancement of the lowest-energy peak.

Fig. 10 presents the imaginary part of the nonlinear optical spectrum *χ*^(3)^_IDIR_(*ω*), corresponding to TPA in the Z20-BPNT, under varying magnetic field strengths. In the unperturbed state, two main TPA peaks appear at 0.49 eV and 0.54 eV, corresponding to transitions between the *E*_11_ and *E*_22_ subbands. Upon the application of an external magnetic field, these peaks exhibit a clear splitting behavior, which arises from the lifting of degeneracy in the related electronic subbands. This field-induced modification is evident in both the real and imaginary components of the *χ*^(3)^_IDIR_(*ω*) spectrum, leading to the emergence of distinct doublet peaks. As shown in Fig. 10(b), the application of a weak magnetic field (*B*_z_ = 0.04) leads to a noticeable splitting of the second peak, whereas the first peak exhibits only a slight splitting accompanied by a distinct red shift and a reduction in intensity. When the field strength is increased to *B*_z_ = 0.08, both peaks clearly split, and the separation between the resulting sub-peaks becomes more pronounced. As shown in Fig. 10(b), a strong field (*B*_z_ = 0.25) induces a remarkable red-shift and a substantial intensity enhancement of the first split peak. This behavior is attributed to the magnetic-field-induced reduction of the energy band gap and enhancement of the corresponding dipole matrix element magnitude.

A similar trend is observed for the real part of the *χ*^(3)^_IDIR_(*ω*) spectrum, as depicted in Fig. 10(c). For magnetic fields exceeding *B*_z_ = 0.08, the negative peaks in the spectrum exhibit evident splitting, with the energy separation between the split components increasing as the field strength grows. Notably, an intensity asymmetry appears between the red- and blue-shifted components, with the red-shifted peak consistently exhibiting higher amplitude, particularly for transitions in the low-energy region *E* < 0.6 eV. This asymmetry arises from the larger dipole matrix elements associated with red-shifted transitions. Under strong magnetic fields (*B*_z_ = 0.25), the overall response intensity increases dramatically [inset Fig. 10(c)], highlighting the strong field-enhancement of nonlinear optical processes in the Z20-BPNT structure.

**Fig. 11:**
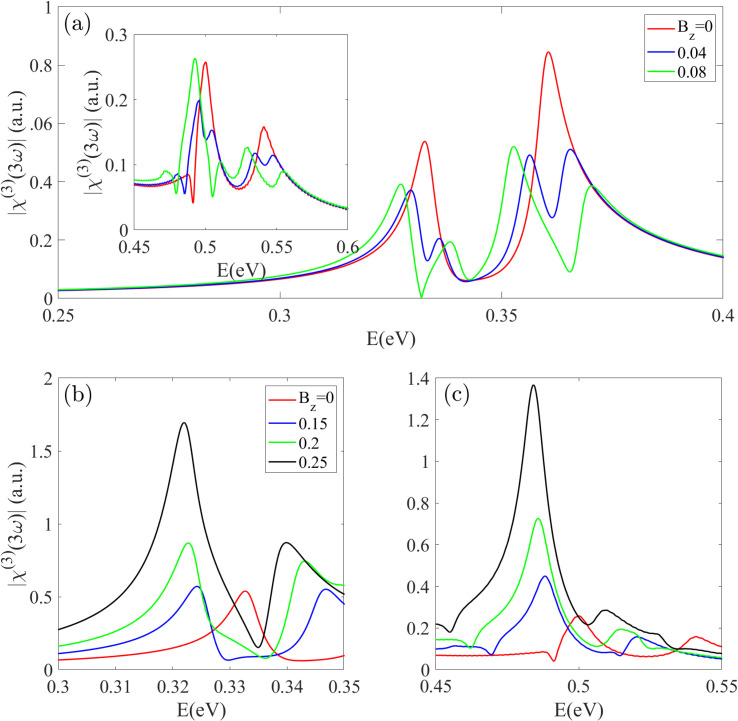
(a–c) The third-harmonic generation spectrum under an increasing magnetic field in the different energy regions. THG peaks located near *E*_g_/3 and *E*_g_/2 progressively split into multiple components as *B*_z_ increases, while stronger fields lead to a pronounced enhancement of their intensities.

Finally, the THG spectrum *χ*^(3)^_THG_(3*ω*) of the Z20-BPNT under different magnetic fields is presented in Fig. 11. The two primary THG peaks (at 0.33 eV and 0.36 eV), originating from three-photon absorption between two nearest subbands to the Fermi level, also undergo splitting due to the lifting of subband degeneracy. The magnitude of this splitting increases proportionally with *B*_z_. For instance, a magnetic field of *B*_z_ = 0.08 splits the first peak into dual peaks at 0.327 eV and 0.338 eV, and the second into components at 0.353 eV and 0.371 eV. With further increases in the magnetic field strength, the split peaks shift over a wider energy range. In the 0.4 < *E* < 0.6 eV region, the Z20-BPNT exhibits two peaks at *E* = 0.5 eV and 0.55 eV, which also experience splitting with noticeable changes in their positions and intensities [inset Fig. 11(a)]. As shown in Fig. 11(c) and (d), a strong field of *B*_z_ = 0.25 leads to a dramatic (∼5-fold) enhancement in the intensity of the primary THG peak, accompanied by a significant red-shift.

Overall, these findings demonstrate that the magnetic field *B*_z_ has a more pronounced effect than the structural parameter *n* in modulating the nonlinear optical properties of BPNTs. The ability to induce and control peak splitting, shifting and intensity variations in both linear and nonlinear spectra, particularly in the infrared region, highlights the potential of BPNTs for tunable optoelectronic applications.

## 4 Conclusions

In this work, a comprehensive theoretical analysis of the linear, nonlinear, and magneto-optical properties of zigzag (*n*, 0) BPNTs within the range 20 ≤ *n* ≤ 28 was conducted using a modified tight-binding and density-matrix approach. The investigation demonstrated that the optical characteristics of these nanostructures are highly dependent on both intrinsic structural parameters and external perturbations. Optical absorption and dielectric spectra exhibit pronounced radius-dependent red- and blue-shifts, enabling structural tuning of the optical response. In the nonlinear regime, BPNTs support strong quadratic electro-optic effects, radius-dependent two-photon absorption, and a distinctive multi-peak third-harmonic generation spectrum not typically observed in carbon nanotubes. More importantly, the study has shown that an axial magnetic field, through the Aharonov–Bohm effect, induces profound changes by lifting the twofold degeneracy of electronic subbands. This leads to a controllable splitting of optical peaks, spectral red-shifts, and a dramatic enhancement of nonlinear signals. These findings highlight that the magnetic field acts as a far more effective modulator of the NLO properties compared to structural parameters. Beyond their scientific novelty, the ability to precisely control optical responses *via* structural parameters and external magnetic fields highlights their potential in next-generation technologies, including nanoscale modulators, quantum switches, and frequency converters.

## Ethics declarations

This manuscript is the authors' original work and has not been published nor has it been submitted simultaneously elsewhere.

## Conflicts of interest

There are no conflicts of interest.

## Supplementary Material

RA-015-D5RA06145H-s001

## Data Availability

The datasets used and analyzed during the current study available from the corresponding author on reasonable request. Supplementary information is available. See DOI: https://doi.org/10.1039/d5ra06145h.
