# Supplementary material for: Linear and nonlinear optical properties of boron phosphide nanotubes: insights into third-harmonic generation and magneto-optical tunability
Source: RSC Adv. 2025 Nov 6;15(50):43141–54. doi: 10.1039/d5ra06145h (PMC12590478; doi:10.1039/d5ra06145h)
Supplement: RA-015-D5RA06145H-s001 [file RA-015-D5RA06145H-s001.pdf]

Supplementary Information (SI) for RSC Advances.
This journal is © The Royal Society of Chemistry 2025

Supporting File

Linear and Nonlinear Optical Properties of Boron Phosphide Nanotubes: Insights into Third-Harmonic Generation and Magneto-Optical Tunability

Raad Chegel[*1]

[1]Department of physics, Faculty of Science, Razi University, Kermanshah, Iran

[1] Email address: Raad.Chegel@gmail.com

DFT calculations:

We performed density functional theory (DFT) simulations using the Spanish Initiative for Electronic Simulations with Thousands of Atoms (SIESTA) package [1]. For the exchange-correlation term, we implemented the Perdew-Burke-Ernzerhof (PBE) functional within the generalized gradient approximation (GGA). A double-ζ plus polarization (DZP) atomic orbital basis set was employed with a plane-wave energy cutoff of 500 Ry. All atomic positions were fully relaxed until the residual force on each atom was less than 0.02 eV/Å. To prevent artificial interlayer interactions, a vacuum spacing of 20 Å was applied along the out-of-plane direction. The Brillouin zone was sampled using a 15×15×1 Monkhorst–Pack k-point grid to ensure convergence. The resulting optimized lattice constant for h-BP is 3.18 Å, which is in excellent agreement with previous DFT reports [2].

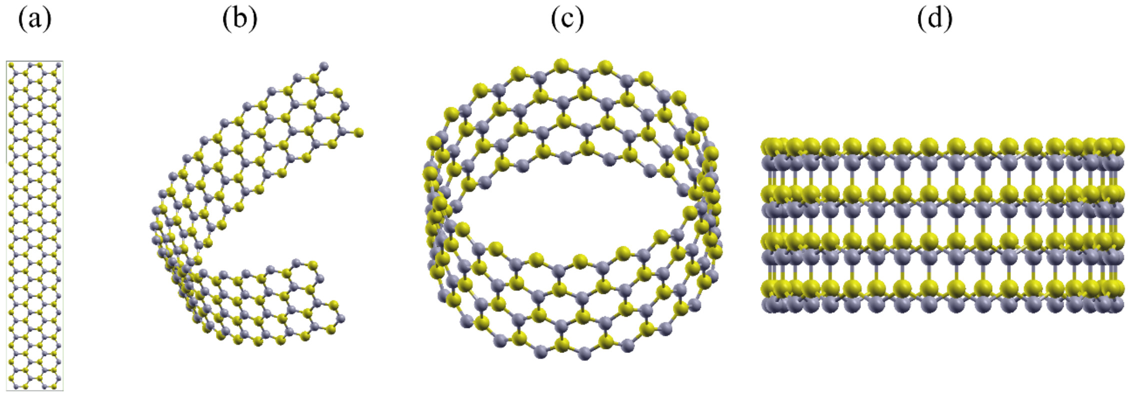

Figure S1. (a) Zigzag BP nanoribbon. (b)–(c) Rolling process of the nanoribbon leading to the formation of a zigzag BP nanotube. (d) The resulting unit cell of the zigzag BPNT, highlighting the arrangement of B and P atoms along the tube axis and demonstrating the structural periodicity in the rolled-up configuration.

Finding the required tight binding parameters for h-BP:

Constructing the tight-binding (TB) Hamiltonian requires determining key parameters, namely the nearest-neighbor hopping integrals and the on-site atomic energies for each atom in the unit cell. For h-BP, we extracted these TB parameters by fitting the TB band structure to results from DFT. To achieve a high degree of accuracy and quantitative agreement, our model was extended to include interactions up to the fifth nearest-neighbor. This approach yields excellent agreement between the TB and DFT band structures for h-BP, as shown in Figure A1, with strong correlation for both the valence and conduction bands across the entire first Brillouin zone.

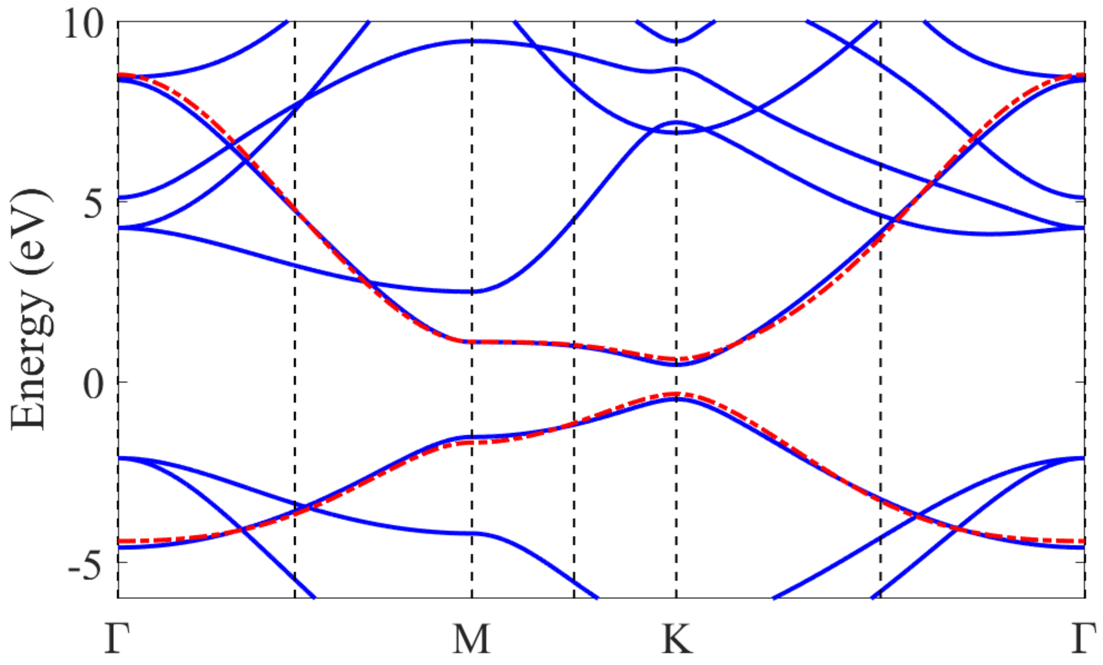

Figure S2. Band structure of monolayer h-BP calculated using the 5NN- tight binding model (red lines) and DFT (blue lines). The tight-binding results exhibit excellent agreement with DFT, especially in the vicinity of the K-point. Both approaches reveal parabolic dispersion of the valence and conduction bands near the K-point and confirm the presence of a direct semiconductor band gap at this high-symmetry point.

Zone-Folding Formalism for BASNTs in the presence of the magnetic field

The electronic structure of BPNTs is derived from that of the monolayer h-BP using the well-established zone-folding approximation [3]. This method imposes periodic boundary conditions on the 2D wave vector, effectively quantizing its component along the nanotube's circumference [$\vec{K}_2$] while preserving its continuous nature along the tube axis [$\vec{K}_1$]. So, the nanotube's wave vector can then be expressed in terms of reciprocal vectors as [3, 4]:

$$k = \mu\vec{K}_1 + k_z\frac{\vec{K}_2}{|\vec{K}_2|} \qquad \mu = 1,\ldots,N \qquad \frac{-\pi}{T} < k_z < \frac{\pi}{T}$$

Here, μ is the discrete subband index, N denotes the number of hexagonal units within the nanotube's translational unit cell, k_z is the continuous axial wave vector and T is the translational period. By substituting this quantized wave vector into the monolayer's dispersion relation, we obtain the 1D subband energy structure for the BPNTs as:

$$E_{BPNT}^{(l)}(k_z,\mu) = E_{h-BP}^{(l)}(\mu\vec{K}_1 + k_z\frac{\vec{K}_2}{|\vec{K}_2|})$$

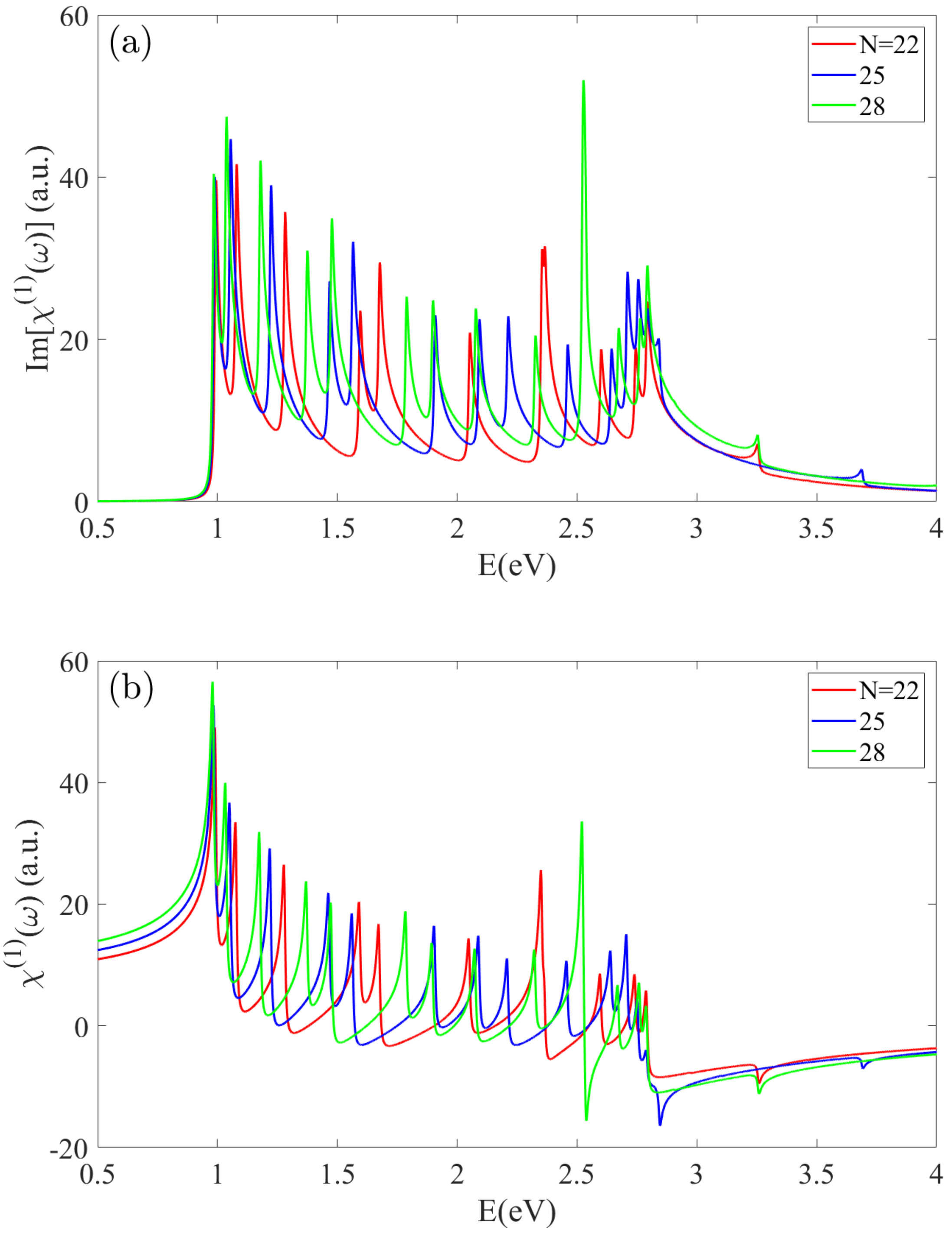

Figure A3. The (a) imaginary and (b) part of the linear optical susceptibility for zigzag BPNTs with S1 family.

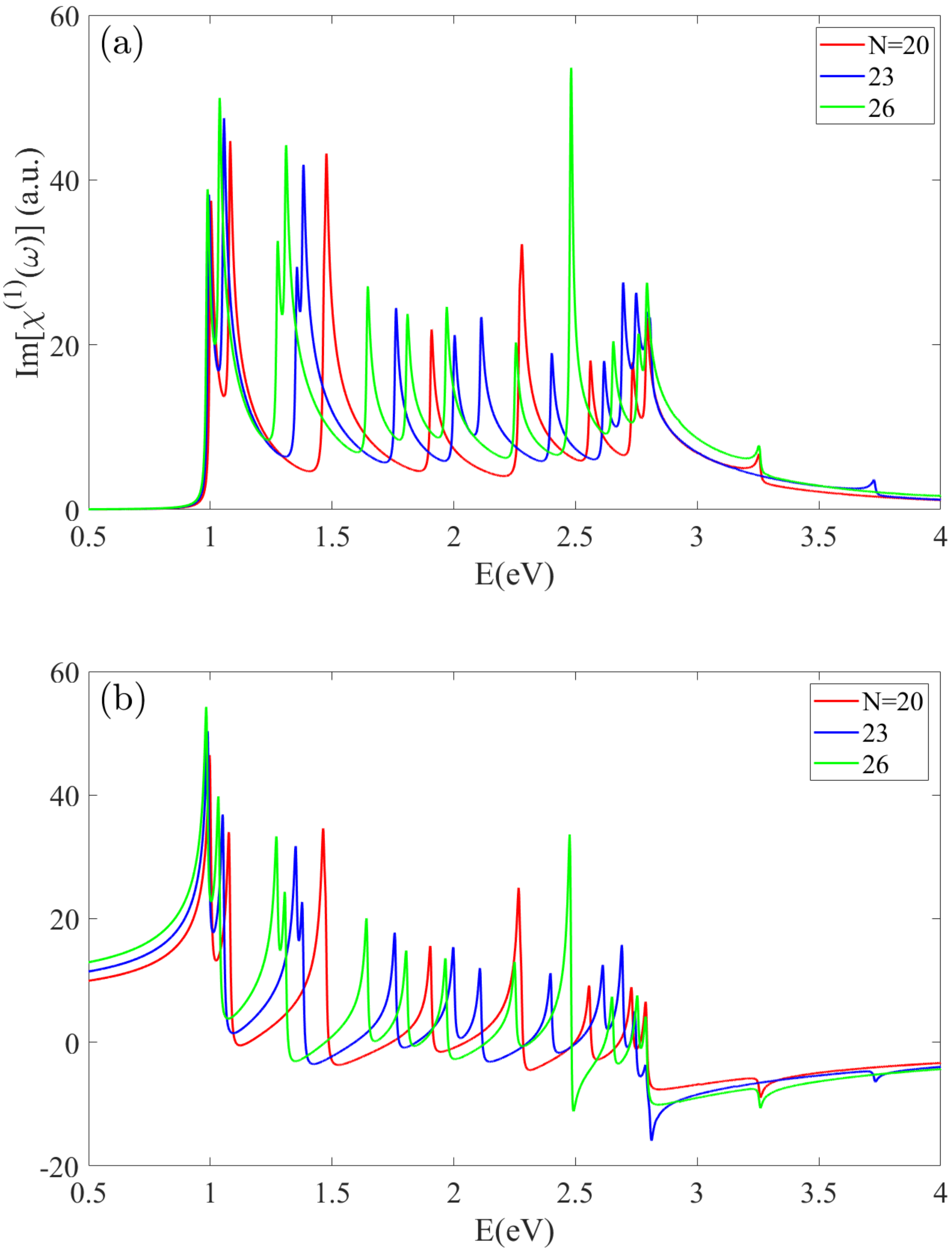

Figure A4. The (a) imaginary and (b) part of the linear optical susceptibility for zigzag BPNTs with S2 family.

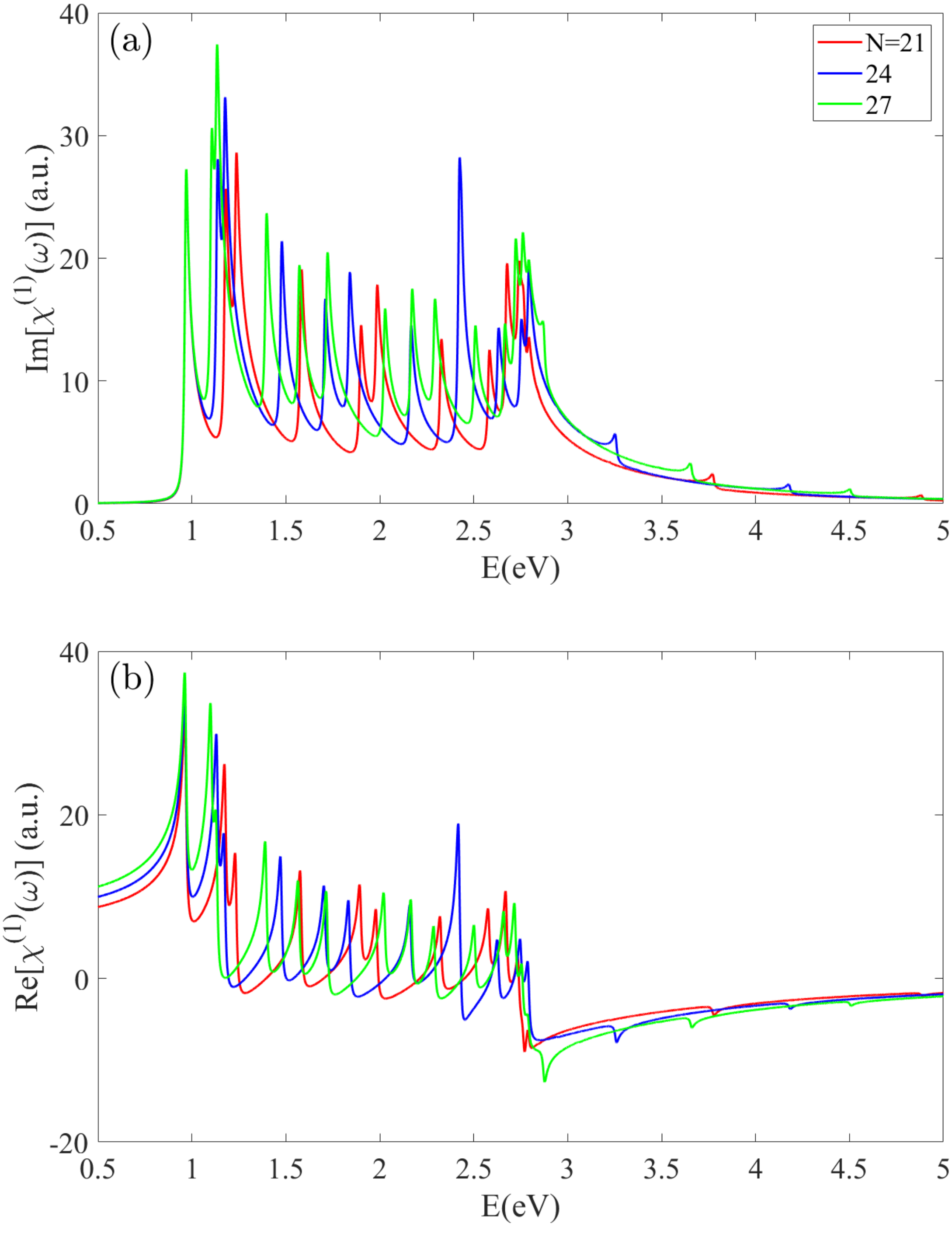

Figure A5. The (a) imaginary and (b) part of the linear optical susceptibility for zigzag BPNTs with S3 family.

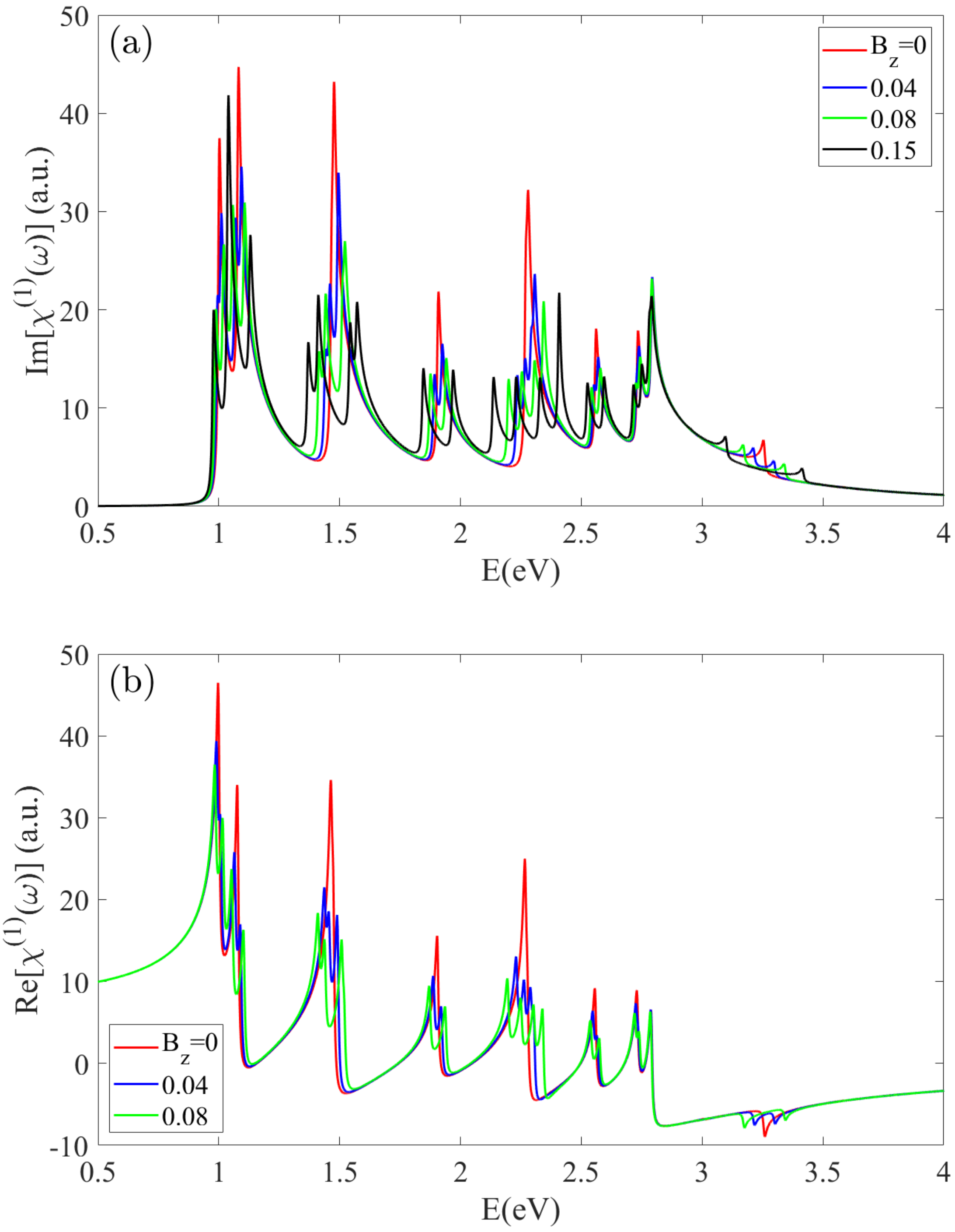

Figure A6. The (a) imaginary and (b) part of the linear optical susceptibility for Z20- BPNT, in the presence of the magnetic field.

[1] J.M. Soler, E. Artacho, J.D. Gale, A. García, J. Junquera, P. Ordejón, D. Sánchez-Portal, The SIESTA method forab initioorder-Nmaterials simulation, Journal of Physics: Condensed Matter, 14 (2002) 2745-2779.
[2] H. Şahin, S. Cahangirov, M. Topsakal, E. Bekaroglu, E. Akturk, R.T. Senger, S. Ciraci, Monolayer honeycomb structures of group-IV elements and III-V binary compounds: First-principles calculations, Physical Review B, 80 (2009) 155453.
[3] R. Saito, M. Fujita, G. Dresselhaus, M.S. Dresselhaus, Electronic structure of chiral graphene tubules, Applied Physics Letters, 60 (1992) 2204-2206.
[4] R. Chegel, A. Zarifi, M. Rezvani Jalal, Chirality and magnetic field engineering of linear and quadratic electro-optic properties in Silicene nanotubes for advanced optoelectronic applications, Physica B: Condensed Matter, 694 (2024) 416435.
